# A Review of Evidence for a Therapeutic Application of Traditional Japanese Kampo Medicine for Oral Diseases/Disorders

**DOI:** 10.3390/medicines5020035

**Published:** 2018-04-18

**Authors:** Marie-Pier Veilleux, Satomi Moriyama, Masami Yoshioka, Daisuke Hinode, Daniel Grenier

**Affiliations:** 1 Oral Ecology Research Group, Faculty of Dentistry, Université Laval, 2420 Rue de la Terrasse, Quebec City, QC G1V 0A6, Canada; marie-pier.veilleux.4@ulaval.ca; 2 Department of Hygiene and Oral Health Science, Tokushima University Graduate School of Biomedical Sciences, Tokushima 770-8504, Japan; moriyama.satomi.1@tokushima-u.ac.jp (S.M.); hinode@tokushima-u.ac.jp (D.H.); 3 Department of Oral Health Science and Social Welfare, Tokushima University Graduate School of Biomedical Sciences, Tokushima 770-8504, Japan; yoshioka.masami@tokushima-u.ac.jp

**Keywords:** Kampo, traditional medicines, herbal medicines, oral diseases, periodontal diseases, mucositis

## Abstract

Kampo medicines prescribed by specialized medical practitioners and Japanese physicians have gradually reemerged in Japan as alternatives to Western medications. Kampo formulations are composed of several plant extracts and, as such, the broad variety of phytochemicals they contain likely act synergistically to provide their beneficial effects. Kampo medicines have traditionally been prescribed for a number of health conditions, including chronic hepatitis, bronchial asthma, anemia, etc. The aim of this article is to review the beneficial effects of Kampos with respect to oral health. Pertinent papers published between 1970 and 2017 were retrieved by searching in PubMed, ScienceDirect, Web of Science, and Scopus using key words followed by evaluation of the relevant articles. In vitro studies have identified a number of properties that give credence to the potential of Kampos for treating or preventing oral diseases/disorders. Given their anti-microbial and anti-inflammatory properties, they may be promising agents for controlling periodontal diseases, oral mucositis, xerostomia, and drug-induced gingival overgrowth. Since some oral diseases have a complex etiology that involves microbial pathogens and the host immune response, agents with dual functionality such as Kampo phytochemicals may offer a therapeutic advantage.

## 1. Introduction

Herbal remedies are traditional medicines developed by combining different parts of local medicinal plants. The use of medicinal plants dates back 5000 years and is part of the cultural heritage of most countries. Traditional Chinese medicine (TCM) developed in China more than 2000 years ago incorporates the use of medicinal plants. To select the appropriate herbal medicines, TCM practitioners diagnose diseases based on a detailed observation of bodily functions, signs, and symptoms. Traditional Japanese medicine, also called Kampo medicine, derives from TCM and appeared around the 5th century A.D. [1,2]. Soon after, Japan began to make modifications to TCM given that some medicinal plants could only be found in China. The recognition by the Japanese government of Kampos as a medical treatment in the 19th century resulted in a standardization of Kampo medicines (mainly herbal extract formulations). The Japanese Ministry of Health, Labor, and Welfare regulates the production of Kampos [3]. Currently, 148 Kampos are reimbursable by the Japanese National Health Insurance Program.

Kampos are prescribed by specialized medical practitioners as well as Japanese physicians who choose to offer traditional alternatives to Western medicine [4,5,6]. Historically, the curative properties of traditional medicines were not supported by rigorous scientific research, especially because they were used to treat the symptoms rather than the cause of the disease. However, many physicians and scientists have become interested in the scientific basis of the effects of traditional medicines. This has led to a vast global effort to comprehensively define the properties of medicinal plants and their natural ingredients. The identification of new medicinal properties of plants may lead to novel approaches for treating modern diseases.

The inclusion of Kampo medicines in the Japanese National Health Insurance Program led them to be strictly standardized by the National Ministry of Health, Labor, and Welfare. A comprehensive list of Kampo regulations is organized and maintained in the Japanese Pharmacopoeia [7]. These regulations control the quality of plant growth, the use and cleanliness of the preparation apparatus, the concoction of the crude drug, and the exact formulation of each Kampo [8].

**Figure 1:**
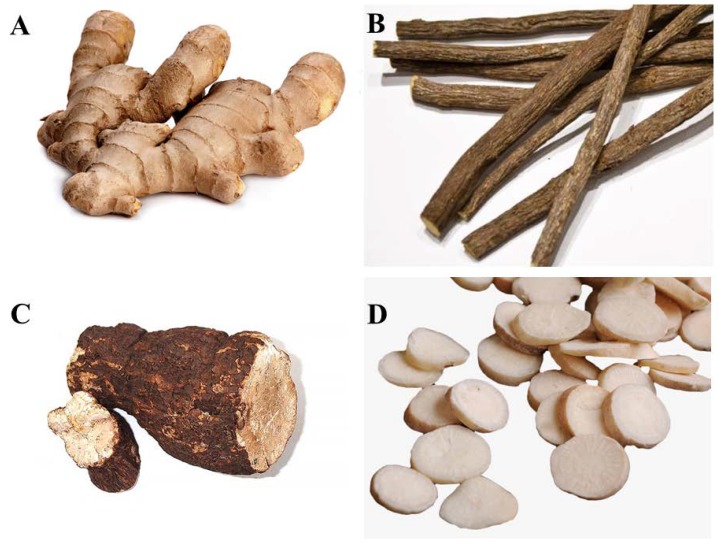
Most commonly used ingredients in Kampo formulations. (**A**) *Zingiber officinale* rhizome (ginger rhizome), (**B**) *Glycyrrhiza uralensis* radix (Chinese licorice root), (**C**) *Poria cocos* (pachyme mushroom), (**D**) *Paeonia lactiflora* radix (Chinese peony root).

Typical Kampos are composed of between five and nine different plants. A number of plants are very commonly used in Japanese Kampos, including *Glycyrrhiza uralensis* radix (Chinese licorice root), *Zingiber officinale* rhizome (ginger rhizome), *Poria cocos* (pachyme mushroom), and *Paeonia lactiflora* radix (Chinese peony root) [9] (Figure 1). For instance, *G. uralensis* radix is found in 94 of 128 Kampo formulas, *Z. officinale* rhizome in 51, *P. cocos* in 46, and *P. lactiflora* radix in 44. In comparison, *Rheum palmatum* rhizome (Chinese rhubarb root) is found in only 16 of the 128 Kampo formulas. While leaves and seeds are sometimes incorporated in the formulas, roots and rhizomes are the most commonly used parts of the plant [9]. Physiologically, the primary function of the leaves is photosynthesis, and the main function of the stems is the transport of nutrients and water. Roots and rhizomes have multiple functions, including nutrient storage and retention. It is thus likely that a greater proportion of active compounds are retained in the root or rhizomes than in any other part of the plant.

Traditionally, medicinal herbs for Kampos were stored in pharmacies in wooden drawers and were manipulated using a special spoon. They were usually administered in the form of a decoction prepared by extracting the plant material with warm water. They could also be used in the form of pills, powders, and ointments. Nowadays, in order to adapt Kampo medicine to modern therapeutic approaches, a variety of preparations of pharmaceutical Kampo extracts have been developed to offer the same effectiveness as the decoction. They are manufactured as granules by lyophilizing the prepared decoction on a large scale, but also by using a process of extraction, solid-liquid separation, concentration, and spray drying [10]. Purified water is always used in the hot water extraction of raw materials while centrifugation is used to separate the residues from the hot water extract. Low temperature vacuum evaporators and spray drying are used to remove the water and minimize thermal denaturation. The use of mixtures of different plants in Kampo formulations results in a greater variety of bioactive compounds in each Kampo. These molecules may interact with each other in complex ways, producing synergistic or even novel effects that are more potent than those of single-plant extracts.

Kampo medicines have traditionally been prescribed for a number of health conditions, including chronic hepatitis, bronchial asthma, allergic rhinitis, anemia, and gastric cancer [11]. For instance, Inchinkoto (TJ-135) is prescribed to treat liver cirrhosis, jaundice, and hepatic inflammation, while Sanoshashinto (TJ-113) is used to treat hypertension, fever, and hypercholesterolemia [9,11]. Evidence for a therapeutic application of Kampo medicines for oral diseases/disorders has also been reported. The aim of this article is to review the beneficial effects of Kampos with respect to oral health. Pertinent papers published between 1970 and 2017 were retrieved by searching in PubMed, ScienceDirect, Web of Science, and Scopus using key words (periodontal diseases, gingival diseases, periodontitis, gingivitis, periodontal inflammation, bone resorption, wound healing, innate immunity, periodontopathogen, periodontal pathogen, oral keratinocyte, oral epithelial cell, gingival fibroblast, periodontal ligament cell, dental plaque, dental biofilm, dental caries, tooth decay, candidiasis, oral mucositis, xerostomia, halitosis, gingival overgrowth, Kampo, traditional Chinese medicine, traditional Japanese medicine) and Boolean operators (AND, OR) followed by evaluation of the relevant articles.

## 2. Kampo Medicines and Periodontal Disease

**Figure 2:**
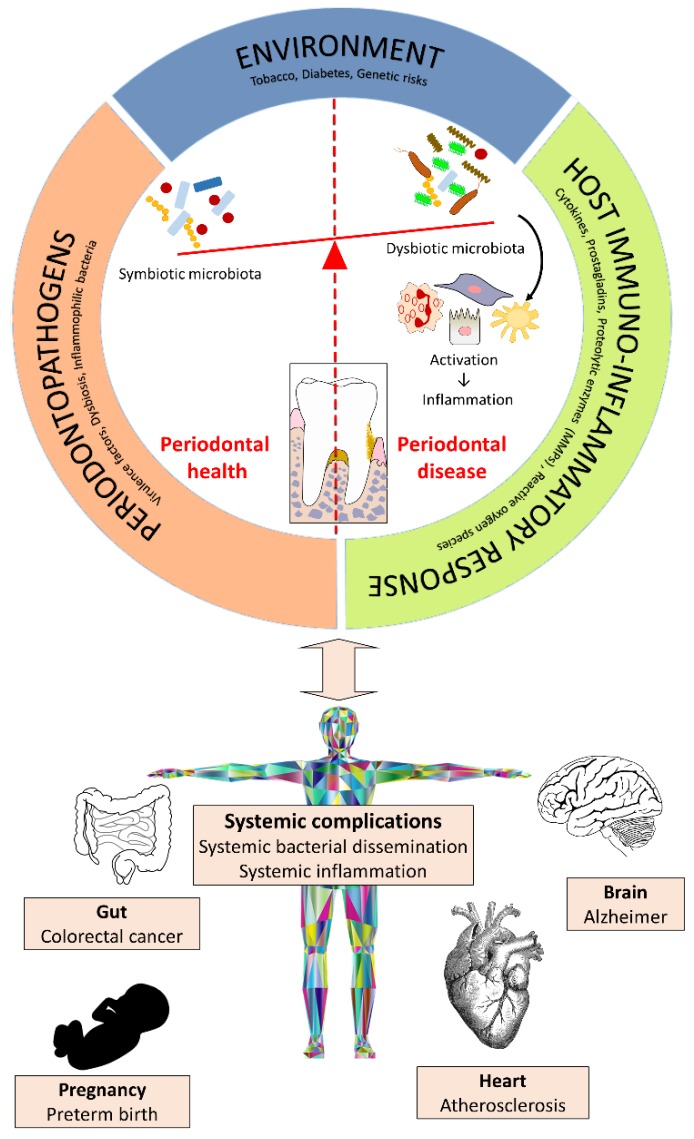
Etiopathogenesis of periodontal diseases and their systemic complications.

The oral cavity harbors one of the most complex microbial ecosystems in the human body. It is estimated that more than 700 bacterial species colonize various sites within the oral cavity. The dental biofilm that develops on the hard and soft tissues of the oral cavity is composed of bacteria, epithelial cells, proteins, enzymes, and food debris, all of which are incorporated in an extracellular polysaccharide matrix [12]. This biofilm initiates periodontal disease that affects the tissues that surround and support the teeth [13]. This condition evolves episodically, with phases of active destruction, latency, and healing. With gingivitis, the inflammatory process is limited to the free gingiva, whereas periodontitis is a progressive disease that affects all tooth-supporting tissues, including the periodontal ligament and the alveolar bone [13]. Two principal factors are involved in the pathogenesis of periodontal disease: the microbial factor, notably the subgingival accumulation of the strictly anaerobic Gram-negative periodontopathogens [14], and the host factor, notably the over-production by resident and immune cells of inflammatory mediators (pro-inflammatory cytokines and prostanoids) and matrix metalloproteinases (MMPs), which can modulate the progression and severity of periodontitis [15,16] (Figure 2). Smoking, diabetes, neutrophil dysfunction, and poor oral hygiene are significant risk factors for periodontitis [17]. Moreover, recent studies have shown that there is an association between periodontal diseases and a variety of systemic complications, including cardiovascular disease, preterm birth, rheumatoid arthritis, and diabetes mellitus [18].

### 2.1. Effect on Periodontopathogenic Bacteria

*Porphyromonas gingivalis*, a late colonizer of the periodontal biofilm, has been strongly associated with the chronic form of periodontitis, where it can be detected in approximately 85% of diseased sites [19]. This Gram-negative anaerobic bacterial species produces a broad array of virulence factors that contribute to tissue colonization and destruction, host defense perturbation, and inflammatory tissue destruction [20]. Liao et al. [21] used a microdilution broth assay to investigate the effect of Kampo formulations on *P. gingivalis*. Of the 27 formulations tested, seven (Tokakujokito [TJ-61], Daiokanzoto [TJ-84], Saoshashinto [TJ-113], Mashiningan [TJ-126], Daijokito [TJ-133], Keishikashakuyakudaioto [TJ-134], and Inchinkoto [TJ-135]) inhibited the growth of *P. gingivalis*, with minimal inhibitory concentrations (MIC) ranging from 250 to 1000 µg/mL. Given that each of these Kampos contained Chinese rhubarb (*Rheum palmatum*), additional antibacterial assays were performed with pure anthraquinones known to be present in rhubarb. Rhein and aloe-emodin displayed the strongest antibacterial activity (MIC = 0.78 µg/mL) against *P. gingivalis* [21]. The seven Kampos with antibacterial properties also reduced the adherence of *P. gingivalis* to oral epithelial cells, but displayed no cytotoxicity at the effective concentrations. Quantitative real-time PCR has shown that Daiokanzoto at sub-MICs inhibits the gene expression of important virulence factors in *P. gingivalis* [22]. More specifically, Daiokanzoto reduces the expression of *fimA* and *hagA*, which are involved in host colonization by *P. gingivalis*. The expression of *rgpA* and *rgpB*, two protease genes associated with the inactivation of host defense mechanisms, tissue destruction, and nutrient acquisition [22] are also down-regulated.

*Fusobacterium nucleatum*, a Gram-negative anaerobic bacterium, is found in higher numbers in subgingival sites affected by chronic periodontitis than in healthy sites [23]. This bacterial species plays a key role in subgingival biofilm formation by bridging the early colonizers (streptococci and actinomyces) and the late colonizers such as *P. gingivalis, Tannerella forsythia,* and *Treponema denticola* [24] that are strongly associated with active periodontal lesions [25]. Given its central role in biofilm formation [24], *F. nucleatum* represents a key target for controlling biofilm formation. Interestingly, Liao et al. [26] showed that Rokumigan (TJ-87), while not affecting the growth of *F. nucleatum*, dose-dependently inhibits the ability of *F. nucleatum* to form a biofilm.

Fukamachi et al. [27] investigated the effect of Hangeshashinto (TJ-14) on the growth of selected oral pathogens. While the growth of Gram-positive bacteria and *Candida albicans* is not inhibited, Hangeshashinto prevents the growth of several Gram-negative periodontopathogenic bacteria, including *P. gingivalis*, *F. nucleatum*, *T. forsythia*, and *T. denticola*. The authors also investigated the effect of purified components from Hangeshashinto and showed that baicalin, berberine, coptisine, [6]-shogaol, and homogentisic acid have antibacterial properties [27].

### 2.2. Effect on the Host Inflammatory Response and Bone Resorption

The host immune response to periodontopathogens, which results in the release of inflammatory mediators by host mucosal and immune cells, mediates localized tissue destruction in periodontitis [15,16]. Consequently, plant-derived compounds endowed with the ability to attenuate the host inflammatory response may have therapeutic properties against periodontal diseases. 

Gingival fibroblasts are the most prominent cells in periodontal tissue [28]. When stimulated by bacterial pathogens or their products such as lipopolysaccharide (LPS), they secrete several pro-inflammatory mediators, including prostaglandin E2 (PGE_2_) [29]. PGE_2_ is a potent inflammatory mediator and has been suggested as a biomarker for diagnosing periodontal disease activity and severity [30]. Two Kampo medicines, Shosaikoto (TJ-9) and Orento (TJ-120), have been shown to attenuate PGE_2_ production by gingival fibroblasts stimulated with LPS [31,32]. Shosaikoto also decreases the activity and blocks the expression of cyclooxygenase-2 (COX-2), a precursor of the inflammatory response [31]. Since PGE_2_ production is regulated by COX enzymes, it has been suggested that Shosaikoto decreases PGE_2_ production by reducing both COX-2 expression and activity [31].

When stimulated with LPS, gingival fibroblasts secrete IL-6 [26], which is a multifunctional cytokine that promotes osteoclast formation and consequently bone resorption [33,34]. While this happens naturally as part of normal bone remodeling, an overproduction of IL-6 may modulate the alveolar bone destruction. Rokumigan (TJ-87) has been reported to inhibit LPS-induced IL-6 secretion by gingival fibroblasts and also attenuate IL-6 secretion by oral epithelial cells [26]. By attenuating IL-6 secretion, Rokumigan may thus contribute to reducing bone resorption. This possibility received support from a study by Shim et al. [35], who provided evidence that *Yukmijihwang-tang* (the Chinese equivalent of Rokumigan) may have therapeutic potential for treating bone diseases by preventing osteoclast differentiation and inhibiting the bone-resorptive activity of differentiated osteoclasts. 

Rikkosan (TJ-110), a Kampo medicine prescribed to control oral pain associated with dental caries, pulpitis, periodontitis, and stomatitis has been reported to exert an anti-inflammatory effect on host cells [36]. More specifically, Horie et al. [36] reported that Rikkosan reduces both IL-1β production by LPS-treated macrophages and PGE_2_ production by IL-1β-treated gingival fibroblasts and periodontal ligament fibroblasts.

Oral epithelial cells produce chemokines, including CXCL8, which are chemoattractants for polymorphonuclear leukocytes and macrophages. Higher levels of CXCL8 are found in the gingival crevicular fluid of inflamed periodontal sites than in healthy sites thus supporting a contribution to periodontitis [37]. Interestingly, periodontal therapy has been shown to reduce immune cell numbers as well as the levels of CXCL8 in gingival crevicular fluid [37]. Daiokanzoto (TJ-84) reduces CXCL8 production by LPS-stimulated oral epithelial cells [22] and may thus contribute to attenuating periodontal inflammation.

In response to various stimuli, including bacterial pathogens, stress, and free radicals, the transcription factor NF-κB is activated and regulates the expression of genes encoding cytokines and MMPs [38]. Given this, inhibiting this signaling pathway has been proposed as an approach for treating periodontal disease [39]. Daiokanzoto, a mixture of crude extracts of Rhubarb rhizomes and Glycyrrhiza roots, has recently been reported to inhibit *P. gingivalis*-induced NF-κB activation in monocytes [22]. Several active constituents of *Glycyrrhiza* spp., including flavanones, chalcones, isoflavans, flavones, and isoflavones, have been shown to inhibit NF-κB activation in various cell types [40].

MMPs are endoproteinases released by several cell types in the periodontium, including fibroblasts, macrophages, and osteoclasts [41]. Since these enzymes can degrade most components of the extracellular matrix, the presence of high levels of MMPs in periodontal sites modulates the destruction of periodontal tissues through degradation of the periodontal ligament, the loss of gingival collagen, and the resorption of alveolar bone [41]. More specifically, high levels of active MMP-9 in gingival crevicular fluid have been associated with periodontal tissue destruction [42], while high levels of MMP-1 mRNA are expressed by periodontitis-affected gingival tissue [43]. Daiokanzoto has been reported to inhibit the catalytic activity of both MMP-1 and MMP-9 and may thus contribute to reducing periodontal tissue damage, including bone resorption [22]. Moreover, Takeda et al. [44] showed the ability of Juzentaihoto (TJ-48) to inhibit osteoclast differentiation in vitro and to reduce alveolar bone destruction in a rat periodontitis model.

### 2.3. Effect on Wound Healing

Wound healing is a complex process involving cell attachment to various components of the extracellular matrix as well as cell migration and proliferation [45]. During wound healing, fibroblasts play a critical role by proliferating and migrating and by remodeling the extracellular matrix by the de novo synthesis of matrix molecules [45]. Rokumigan has been reported to have a positive effect in a wound healing model by dose-dependently enhancing the migration of gingival fibroblasts [26]. These effects may be of interest for the management of gingival wounds associated with periodontal diseases.

### 2.4. Effect on the Innate Immunity of Epithelial Cells

The epithelial barrier is the first line of defense against several microbes providing physical and chemical protection [46]. Moreover, epithelial cells can produce different antimicrobial peptides, including calprotectin, defensins, and cathelicidin, which have a broad spectrum of activity against bacteria, fungi, and some viruses. These antimicrobial peptides, some of which also possess anti-inflammatory properties, are thought to contribute to periodontal health [47]. Shosaikoto (TJ-9) and Hangeshashinto (TJ-14) up-regulate the expression of calprotectin in oral epithelial cells [48,49], most likely through an IL-1α-mediated pathway. These results suggest that these Kampos may contribute to controlling infections in the oral cavity and may thus be potential candidates for the prevention and treatment of periodontal diseases.

## 3. Kampo Medicines and Oral Mucositis

Oral mucositis is characterized by a painful and ulcerous inflammation of the oropharyngeal mucosa [50]. Although sometimes asymptomatic, patients can experience burning, stinging, taste changes, and even pain that may prevent them from eating, especially patients with head or neck cancer who receive chemotherapy or radiotherapy treatments [50]. Mucositis can also develop due to the effect of anticancer drugs (irinotecan, fluorouracil) on mucosal cells [50]. Ulcerative mucositis may be further complicated by local infections by viruses, fungi, or bacteria. Currently, for the majority of patients, no effective interventions are available. Therefore, there is a need for developing novel strategies for the prevention and treatment of mucositis.

Yoshida et al. [51] recently showed that Daiokanzoto can attenuate cell death induced by 5-fluorouracil through inhibition of mitochondrial reactive oxygen species production by gingival cells. This led to the suggestion that Daiokanzoto could be of interest to treat oral mucositis in patients receiving multicycle chemotherapy.

Previous studies reported that the repeated topical application (mouthrinse, cotton pellet) of Hangeshashinto to mucositis lesions improved the severity of symptoms, including pain and oral intake difficulty, in the majority of patients [52,53]. Evidence was found suggesting that Hangeshashinto is acting by suppression of cyclooxygenase-2 expression and prostaglandin E_2_ activity in oral keratinocytes as well as chemotaxis of inflammatory cells [54,55]. Active ingredients in Hangeshashinto were identified as [6]-shogaol, [6]-gingerol, wogonin, baicalein, baicalin, and berberine [54].

## 4. Kampo Medicines and Xerostomia

Xerostomia, also known as dry mouth syndrome, is often caused by anticholinergic drugs, antihistamines, antipsychotics, Sjogren’s syndrome, and diabetes that reduce saliva secretion [56]. Saliva, through its antimicrobial, lubricating, and buffering properties, plays a critical role in maintaining oral health. Consequently, a reduction in saliva flow can predispose to various oral disorders such as caries, periodontal disease, and halitosis. 

Byakkokaninjinto (TJ-34) was found to stimulate the secretion of saliva in rats in a dose-dependent manner [57]. Byakkokaninjinto also significantly improved the secretion of saliva by mice that have been pre-treated with drugs causing mouth dryness, including propranolol (βα-adrenergic blocker), phentolamine (α-adrenergic blocker), 4-DAMP (selective M3 muscarinic receptor blocker), or atropine (anti-adrenergic blocker) [58]. Similar observations were also obtained with the Kampo formula Goreisan (TJ-17) [59].

## 5. Kampo Medicines and Drug-Induced Gingival Overgrowth

Several drugs, including nifedipine, a calcium channel antagonist, phenytoin, an antiepileptic, and cyclosporin, an immuno-suppressor, can cause the overgrowth of gingival fibroblasts in approximately half of the people who take these agents [60]. Gingival overgrowth causes a major problem for the maintenance of oral hygiene. Moreover, the increased swelling of gingiva promotes the risk of bacterial infections [60]. Gingivectomy, that is, surgically removing excess gingival tissue, is the current treatment for this condition. There is currently no treatment to prevent gingival overgrowth.

Saireito (TJ-114), a Kampo used to treat glomerulonephritis, nephrotic syndromes, and diabetic nephropathies, inhibits the proliferation of mesangial cells [61]. Based on this effect, it has been suggested that Saireito may also inhibit the growth of gingival fibroblasts induced by nifedipine, a calcium antagonist. Hattori et al. [62] showed that Saireito inhibits the nifedipine-induced proliferation of gingival fibroblasts in a dose-dependent manner. This appeared to be associated with the ability of Saireito to reduce the release of nifedipine-induced bFGF and the production of type I collagen [62]. The effect of Saireito was also examined in a nifedipine-induced gingival overgrowth in a rat model. More specifically, this Kampo suppressed nifedipine-induced expansion of the interval between mandibular incisors and the hyperplasia of oral gingiva at the maxillary first molar. These results strongly suggest that Saireito could be useful to treat gingival overgrowth induced by medications.

## 6. Conclusions

Traditional Japanese Kampo medicine, which is covered by the Japanese National Health Insurance Program is used by medical doctors in Japan as a part of their regular practice. The diagnostic and treatment procedures for Kampo are completely different from those of modern Western medicine. Kampo formulations are used to treat a wide variety of conditions, including gynecological problems, allergies, rheumatoid arthritis, chronic hepatitis, diabetic retinopathy, bronchial asthma, and high cholesterol levels.

Since some oral diseases/disorders have a complex etiology, agents with the ability to act on several targets such as Kampo phytochemicals may offer a therapeutic advantage (Table 1). In vitro studies have recently identified a number of properties that give credence to the potential of Kampos for treating or preventing oral diseases. Given their anti-adherence, anti-microbial, and anti-inflammatory properties, they may be promising agents for controlling oral diseases.

## Figures and Tables

**Table 1:** List of Kampos with potential therapeutic properties for oral diseases/disorders.

Name of Kampo	Type of Studies	Disease/Disorder	Effect of Kampo	Reference
Byakkokaninjinto (TJ-34)	In vivo (animal)	Xerostomia	Stimulation of saliva secretion	[57,58]
Daijokito (TJ-133)	In vitro	Periodontal disease	Growth inhibition of periodontopathogens	[21]
Daiokanzoto (TJ-84)	In vitro	Periodontal disease	Growth inhibition of periodontopathogens Inhibition of bacterial virulence factor gene expression Inhibition of LPS-induced CXCL8 production by oral epithelial cells Inhibition of periodontopathogen-induced NF-κB activation in monocytes Inhibition of MMP-1 and MMP-9 catalytic activity	[21]
				[22]
				[22]
				[22]
				[22]
	In vitro	Oral mucositis	Inhibition of 5-fluorouracil-induced gingival cell death	[51]
Goreisan (TJ-17)	In vivo (animal)	Xerostomia	Stimulation of saliva secretion	[59]
Hangeshashinto (TJ-14)	In vitro	Periodontal disease	Up-regulation of calprotectin expression in oral epithelial cells	[49]
			Growth inhibition of periodontopathogens	[49]
	In vivo (human)	Oral mucositis	Improvement of mucositis lesions (topical application)	[27]
	In vitro	Oral mucositis	Inhibition PGE_2_ production by oral keratinocytes	[52,53]
			Inhibition of cyclooxygenase-2 expression and chemotaxis in inflammatory cells	[54,55]
Inchinkoto (TJ-135)	In vitro	Periodontal disease	Growth inhibition of periodontopathogens	[21]
Juzentaihoto (TJ-48)	In vitro	Periodontal disease	Inhibition of osteoclast differentiation	[44]
	In vivo (animal)	Periodontal disease	Reduction of alveolar bone destruction	[44]
Keishikashakuyakudaioto (TJ-134)	In vitro	Periodontal disease	Growth inhibition of periodontopathogens	[21]
Mashiningan (TJ-126)	In vitro	Periodontal disease	Growth inhibition of periodontopathogens	[21]
Orento (TJ-120)	In vitro	Periodontal disease	Inhibition of LPS-induced PGE_2_ production by gingival fibroblasts	[32]
Rikkosan (TJ-110)	In vitro	Periodontal disease	Inhibition of LPS-induced PGE_2_ production by gingival fibroblasts	[36]
			Inhibition of IL-1β-induced PGE_2_ production by gingival fibroblasts and periodontal ligament fibroblasts	[36]
Rokumigan (TJ-87)	In vitro	Periodontal disease	Inhibition of biofilm formation by periodontopathogens	[26]
			Inhibition of LPS-induced IL-6 production by gingival fibroblasts and oral epithelial cells	[26]
			Enhancement of gingival fibroblast migration (wound healing)	[26]
Saireito (TJ-114)	In vitro	Drug-induced gingival overgrowth	Inhibition of nifedipine-induced gingival fibroblast overgrowth	[62]
			Inhibition of type I collagen production by gingival fibroblasts	[62]
Saoshashinto (TJ-113)	In vitro	Periodontal disease	Growth inhibition of periodontopathogens	[21]
Shosaikoto (TJ-9)	In vitro	Periodontal disease	Inhibition of LPS-induced PGE_2_ production by gingival fibroblasts	[31]
			Up-regulation of calprotectin expression in oral epithelial cells	[48]
Tokakujokito (TJ-61)	In vitro	Periodontal disease	Growth inhibition of periodontopathogens	[21]
